# Comparison between Accelerometer and Gyroscope in Predicting Level-Ground Running Kinematics by Treadmill Running Kinematics Using a Single Wearable Sensor

**DOI:** 10.3390/s21144633

**Published:** 2021-07-06

**Authors:** Daniel Hung Kay Chow, Luc Tremblay, Chor Yin Lam, Adrian Wai Yin Yeung, Wilson Ho Wu Cheng, Peter Tin Wah Tse

**Affiliations:** 1 Department of Health & Physical Education, The Education University of Hong Kong, Hong Kong, China; waiyyeung@eduhk.hk (A.W.Y.Y.); hwcheng@eduhk.hk (W.H.W.C.); ptwtse@eduhk.hk (P.T.W.T.); 2 Faculty of Kinesiology & Physical Education, University of Toronto, Toronto, ON M5S 2W6, Canada; luc.tremblay@utoronto.ca; 3 Department of Orthopaedics & Traumatology, Li Ka Shing Faculty of Medicine, The University of Hong Kong, Hong Kong, China; lam_clive_cy@post.harvard.edu

**Keywords:** deep learning, convolutional neural network, running, kinematics, wearable sensor, running kinematics analysis, accelerometer, gyroscope

## Abstract

Wearable sensors facilitate running kinematics analysis of joint kinematics in real running environments. The use of a few sensors or, ideally, a single inertial measurement unit (IMU) is preferable for accurate gait analysis. This study aimed to use a convolutional neural network (CNN) to predict level-ground running kinematics (measured by four IMUs on the lower extremities) by using treadmill running kinematics training data measured using a single IMU on the anteromedial side of the right tibia and to compare the performance of level-ground running kinematics predictions between raw accelerometer and gyroscope data. The CNN model performed regression for intraparticipant and interparticipant scenarios and predicted running kinematics. Ten recreational runners were recruited. Accelerometer and gyroscope data were collected. Intraparticipant and interparticipant R^2^ values of actual and predicted running kinematics ranged from 0.85 to 0.96 and from 0.7 to 0.92, respectively. Normalized root mean squared error values of actual and predicted running kinematics ranged from 3.6% to 10.8% and from 7.4% to 10.8% in intraparticipant and interparticipant tests, respectively. Kinematics predictions in the sagittal plane were found to be better for the knee joint than for the hip joint, and predictions using the gyroscope as the regressor were demonstrated to be significantly better than those using the accelerometer as the regressor.

## 1. Introduction

Although running is a popular recreational sport worldwide, injury incidence rates ranging from 19.4% to 79.3% have been reported [1]. These injuries are mostly due to overuse of the lower extremities. Among various lower extremity injuries, injuries in the region surrounding the knee were found to be the most common and have severe consequences [1], such as patellofemoral pain syndrome, anterior cruciate ligament injury, iliotibial band friction syndrome, and patellar tendinopathy [2,3]. Therefore, the investigation of lower extremity kinematics is important for the better understanding of running-related injuries.

In human running kinematics analysis, a camera-based motion capture system is considered the gold standard [4]. The capture system is constructed in a laboratory. The research participant is surrounded by cameras and retroreflective markers are placed on anatomical landmarks of the human body [4]. Despite the use of multiple cameras, a body part or object can occlude the field of view between a marker and the cameras, decreasing the effectiveness of such motion tracking systems. In addition, despite the valid and reliable measurements provided by such a system, the technology is confined to the laboratory setting and requires expensive, sophisticated equipment that is permanently installed in a large room [5,6,7]. Therefore, although camera-based motion capture systems are the gold standard, making valid assessments of level-ground running kinematics using these systems is still challenging.

With technological advancements in sensors and data analysis, real-time mobile sensor devices have been developed. Wearable, mobile sensors can be used to analyze human gait patterns in the real world and in outdoor running environments [8]. Inertial measurement units (IMUs)—including accelerometers, gyroscopes, and magnetometers—have been employed extensively in recent studies to detect acceleration and angular velocity [9,10,11,12,13]. Human gait kinematics, such as that pertaining to joint angles, can be estimated by merging inertial data from two body segments [14,15,16]. Compared with camera-based motion capture systems, wearable sensors are more portable and can monitor limb kinematics in the field without obstructing normal gait [5].

Numerous studies have examined the validity and reliability of using wearable sensors for joint angle estimation during level walking [17,18,19,20] and running [18,21,22,23]. Typically, joint angle assessment requires at least two IMU sensors, with each placed on one body segment of the joint studied [24,25,26]. Therefore, multiple sensors are required. Usually, 18 sensors are used in total, with 11 sensors placed on the upper body and 7 placed on the lower extremities [16,27,28,29,30,31].

To reduce redundancy and cost, a study attempted to minimize the number of sensors used during human kinematics analysis [32]. Two approaches have been proposed to reduce the number of sensors, namely, the model-based approach [33,34,35] and the data-driven approach [5,36,37]. The model-based approach involves establishing a serial kinematic chain model and calculating inverse kinematics to estimate the motion of the lower extremities [5,35]. Several studies have investigated lower extremities kinematics with reduced numbers of IMU sensors [33,34,35]. A study has used a single IMU sensor to detect age- and surface-related differences in walking with machine learning algorithms [38]. However, model-based approaches have also been criticized for errors arising from the misalignment of the sensors during the set-up or the trial (see [5,39]).

By contrast, the data-driven approach employs supervised machine learning models to estimate lower extremity kinematics [5,36]. Numerous studies have applied the machine learning method to predict extremities kinematics during running or walking [32,33,36,37,40]. Zimmermann et al. [26] determined the time dynamic features of lower body kinematics for improved orientation alignment and IMU-to-segment assignment tasks by using a deep learning approach that included CNNs combined with long short-term memory networks and generalized recurrent units. Lastly, Gholami et al. [5] developed a novel method using a single shoe-mounted accelerometer and CNN to estimate lower extremity gait kinematics in the sagittal plane during treadmill running. These data-driven approaches minimize the number of IMUs while maximizing the accuracy of the measurements by using supervised machine learning models.

Different types of IMUs, namely accelerometers and gyroscopes, have been used to estimate human gait kinematics with differing performance [14,15,16]. Rhudy and Mahoney [41] reported that estimations in step counting were better when using gyroscopic sensors than when using accelerometer sensors. Mahoney and Rhudy [42] also presented a machine learning method in gait stride categorization (i.e., walking, jogging, or running). Artificial neural network models trained with raw accelerometer data performed better (specifically, categorizing gait stride more accurately) than those trained using gyroscopic data (see [42]). These results demonstrate that different sensors have different advantages for encoding level-ground gait characteristics. However, performance in predicting running kinematics using accelerometers or gyroscopes is unconfirmed.

These studies [5,8,10,11,12,15,16,17,18,19,20,21,22,23,24,26,27,28,29,30,31,32,33,34,35,36,37,38,39,40] have pertained to the use of camera-based motion analysis systems to capture target kinematics. The myoMotion (Noraxon, Scottsdale, USA) sensors are a set of sensors for specific body locations (i.e., 7 sensors for the lower extremities or 16 sensors for the full body), whereas IMeasureU (Vicon, Oxford, UK) sensors can be used individually and independently to capture raw data at a given location. The attachment of the IMeasureU sensor at the anteromedial tibia is also more convenient for users. Because IMUs have been shown to be reliable and accurate for use outside of laboratory settings, recent studies have aimed to reduce the number of sensors worn, with the ultimate aim of using only a single IMU, to measure running kinematics. This study used two sets of IMUs: one (myoMotion) was used to capture target joint kinematics, whereas another one (IMeasureU) was used to capture regressor data for modeling the target joint kinematics using a deep learning approach. This study aimed to (1) use a CNN model to predict level-ground running kinematics, measured by four IMUs on the lower extremities of the right side, using treadmill running kinematics training data, measured using a single IMU on the anteromedial side of the right tibia and (2) compare the performance of level-ground running kinematics predictions between raw accelerometer and gyroscope data.

## 2. Materials and Methods

### 2.1. Participants

Five male and five female healthy recreational runners (age: 22.70 ± 1.34 years, height: 168 ± 6.32 cm, and weight: 61.33 ± 6.82 kg) were recruited to participate in this study. The participants had no history of injury within the previous 6 months. Written informed consent (approved by the Human Research Ethics Committee of the University) was obtained from each participant before data collection.

### 2.2. Instruments

**Figure 1:**
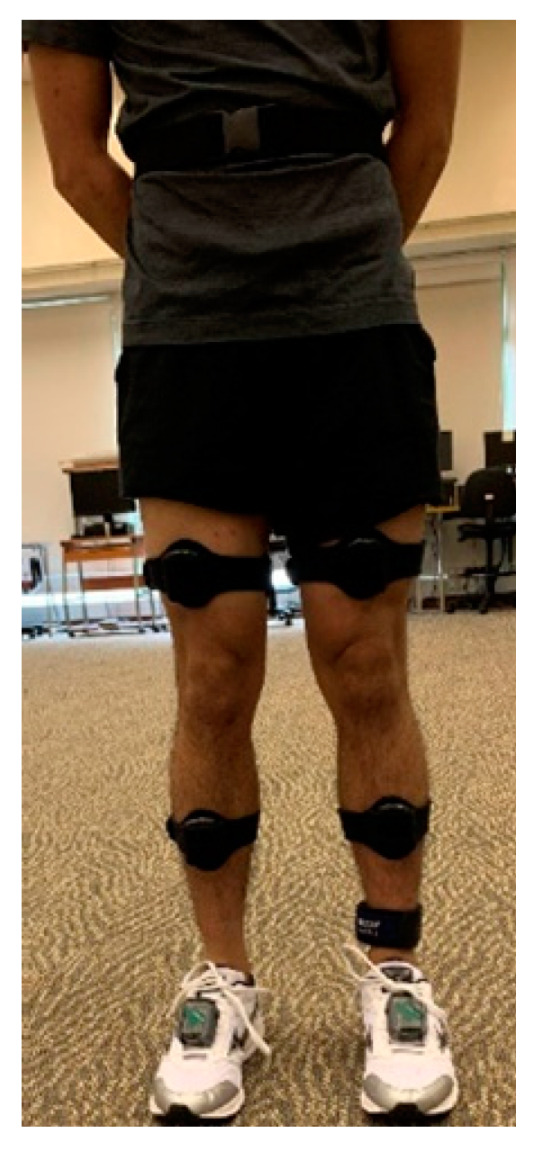
The sensors’ placement on the participant.

**Figure A1:**
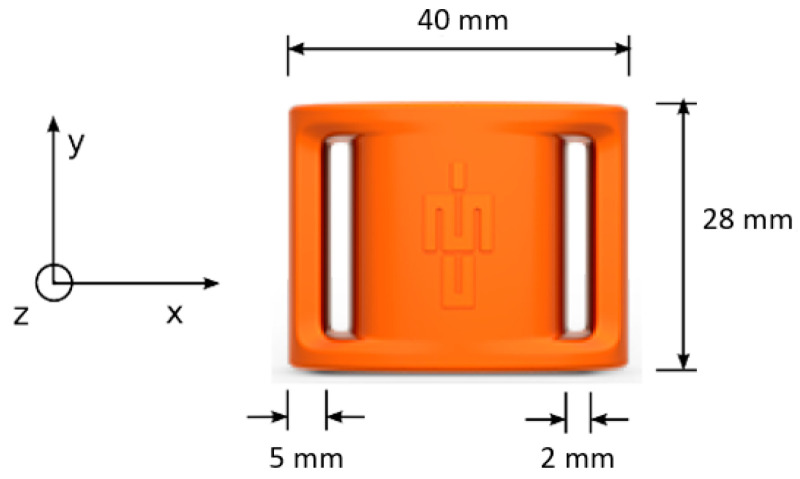
Directions of *x*, *y*, and *z* axes on the IMeasureU sensor (Vicon, Oxford, UK).

**Figure A2:**
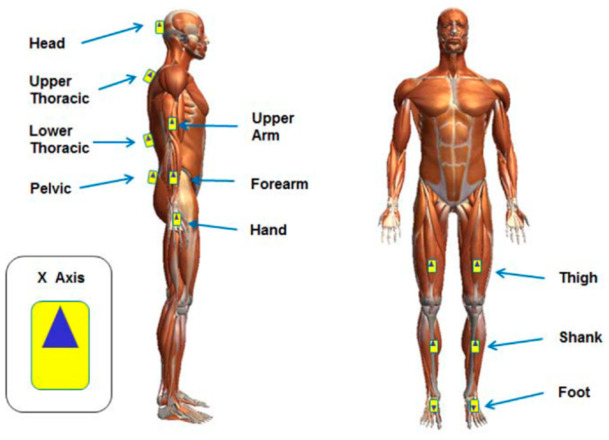
The sensors’ placement and direction of *x* axis of myoMotion sensors (Noraxon, Scottsdale, AZ, USA).

All participants were equipped with the two sets of IMUs. Specifically, myoMotion sensors were used to capture lower limb movement. According to the sensor placement for lower extremities recommended by the manufacturer (Noraxon), seven myoMotion sensors were attached to the participants with either an elastic strap or belt on the pelvis, left and right thighs, left and right shanks, and left and right feet. The sensors’ placement is indicated in Figure 1. Only data collected by the four sensors attached on the pelvis and right leg were considered and used for data analysis. Accelerations (on the *x*, *y*, and *z* axes) and anatomical angles for the lower limbs (knee flexion/extension and hip flexion/extension) were measured with a sampling rate of 100 Hz. Second, a single IMeasureU sensor was attached to the anteromedial side of the right tibia, and tri-axial accelerations and angular velocities were recorded. The raw data of the accelerometer and gyroscope were acquired, at a sampling rate of 500 Hz, and used for data processing. The two sets of sensors were synchronized with three vertical right foot strikes at the beginning of each trial [40]. The directions of the axes of IMeasureU and myoMotion are indicated in Appendix A Figure A1 and Figure A2, respectively.

### 2.3. Experiment Protocol and Data Collection

All participants were required to complete the Physical Activity Readiness Questionnaire (PAR-Q, Canadian Society for Exercise Physiology, 2002. www.csep.ca/forms, 30 September 2020) and a medical history questionnaire before the study began. Those with a “Yes” to one or more questions in the PAR-Q, or with any obvious anatomical abnormalities, were excluded. Anthropometric data, including height, weight, and age, were measured and collected. All running trials were conducted on a treadmill in the laboratory. Participants were advised to wear their usual running attire with self-selected running shoes and that they should not participate in any physical activity on the day prior to testing. The IMeasureU and myoMotion sensors were equipped, calibrated, and synchronized. After synchronization, participants warmed up at 1.5 m/s on the treadmill for 30 s. Every 3 min, the speed of the treadmill was increased, first to 2.0 m/s, then to 2.5 m/s, to 3.0 m/s, and finally to 3.5 m/s [5,43]. The participants were then asked to run in an indoor squash court for 3 min at their preferred speed (average speed: 2.44 ± 0.34 m/s), which was recorded using a Brower Timing System (Brower Timing Systems, Draper, UT, USA). The data obtained from the two sets of IMUs were compared.

### 2.4. The Deep Learning Regression Model

The CNN deep learning model used by Gholami et al. [5] was used to compare the results. Gholami et al. [5] used four layers of one-dimensional convolutional layers (Conv1D) to model the target variable array Y containing the hip, knee, and ankle flexion values. A four-column array X was constructed with the first three columns corresponding to the orthogonal accelerations (a*_x_*, a*_y_*, and a*_z_*) and the last column corresponding to the magnitude of the total accelerations, a*_xyz_*. The rows of X correspond to the IMU data collected at 100 Hz. X was separated into overlapping frames of dimension 60 × 4, representing 0.6 s of IMU data, and a time window was applied to each frame to determine Y at time t by considering the frame representing the 0.6 s of data from t −0.3 s to t +0.3 s.

The same parameters used by Gholami et al. [5] were used in this study. Specifically, the first two Conv1D layers had 50 filters followed by a maximum pooling layer of 2:1 subsampling. The following two Conv1D layers had 100 filters, and the outputs were flattened and fed into a dense layer of 100 neurons before being consolidated into Y with 3 neurons. A kernel size of 3 with a stride value of 1 and a rectified linear unit, abbreviated as ReLU [44], was used for activation in all layers except the regression output. For optimization, Adam [45] was used with a learning rate of 0.001 and batch size of 512 at 50 epochs to achieve rather a stable convergence. For the initialization of neuron weights, the Glorot normal initializer [46] was used (also called the Xavier normal initializer). The Python 3.7 with the Tensorflow and Keras packages were used for implementation.

Different arrangements of regressors for X and targets for Y were applied in our study. Since accelerometer readings were likely to deform to different extents on different running surfaces, the gyroscope was also used as a regressor because of the readings being less subject to such deformations. For the accelerometer, the y-component, which was more or less aligned with the tibial direction, was used as X. For the gyroscope, the resultant of the x-component and the z-component was used, which more or less reflected the angular motions on the sagittal plane. This selection of regressors was to minimize variations in sensor orientation during the experiments. While Gholami et al. [5] used a combined loss function for the hip, knee, and ankle and used a set of weights to favor hip and ankle optimization in the regression, the three targets were separated for the best optimization results in this study. Therefore, while X and Y were univariate in our case and multivariate in Gholami et al. [5], the subsequent arrangement of X and Y as tensor arrays for training was the same. Furthermore, to achieve similar convergence using a fixed 50 epochs, the gyroscope data were scaled down by 100 times to a similar numerical range as the accelerometer values. The parameters of the CNN network used in this study are summarized in Table 1.

### 2.5. Evaluation Methods

In the present study, the CNN was adopted to evaluate two (intraparticipant and interparticipant) scenarios. For the intraparticipant CNN model, the model was trained on each participant’s treadmill running data separately and tested on that participant’s level-ground running data. To obtain steady-state data, the first 15 s and last 15 s of the treadmill running data from each participant at every speed were designated as the ingress buffer and not included in the data analysis. The remaining treadmill running data (2.5 min) of each participant at every speed were then segmented into three sets of 60, 60, and 30 s, in which the first set of 60 s at each speed was extracted and concatenated as training data (4 min total data, 1 min for each running speed). The level-ground running data of each participant were treated similarly to extract test data. The ingress buffer was discarded, the remaining 2.5 min was fragmented into three sets of 60, 60, and 30 s, and the first set of 60 s was extracted and used as test data.

For the interparticipant evaluation, a leave-one-out scheme similar to that in Gholami et al. [5] was adopted for analysis. With the same data fragmentation and assignment of training and test data sets as those in the intraparticipant CNN model, the treadmill running data of nine participants were used to train the model, and the data of the 10th participant were used to test the model’s predictions. These training and testing procedures were performed 10 times with each participant designated as the left-out participant once.

Six parameters (Table 2) calculated using algorithms in Python 3.7 were used for the analysis in the current study: (1) average correlation (R^2^), (2) average root mean squared error (RMSE), (3) average normalized root mean squared error (NRMSE), (4) average standard deviation (STD), (5) average normalized standard deviation (NSTD), and (6) average range of motion (ROM). The average R^2^ reflects the goodness-of-fit of a regression model. The average RMSE and NRMSE represent differences between actual and predicted values. The average STD and NSTD indicate the amount of variation or dispersion in a model. The average ROM is the overall movement of the hip and knee joints in the sagittal plane.

**Figure 2:**
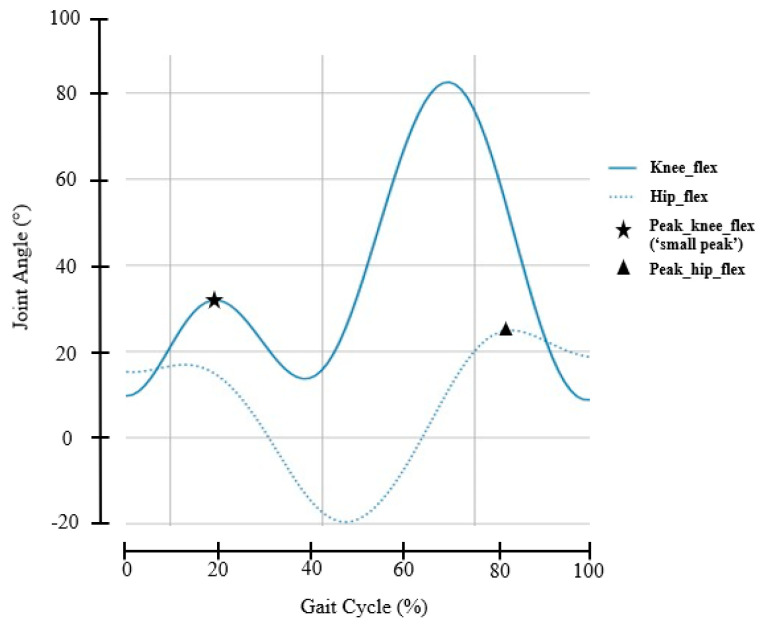
Example data segment showing peak detections of knee and hip joint angles in the sagittal plane measured using myoMotion.

Following Gholami et al. [5], in the calculation of RMSE, NRMSE, STD, and RSTD as shown in Table 2, the peaks’ positions indicated in Figure 2 were used, i.e., the ‘small peak’ for the knee flexion and the maximum position in the hip flexion. For R^2^, however, all points in the curves were used.

### 2.6. Statistical Analysis

Paired sample *t*-tests were used to compare the average R^2^, average RMSE, average NRMSE, average STD, and average NSTD between an accelerometer regressor and a gyroscope regressor. The associated interpretation of the R^2^ was used in accordance with Schober et al. [47]. Correlation coefficient with 0–0.10, 0.10–0.39, 0.40–0.69, 0.70–0.89 and 0.90–1.00 were interpreted as negligible correlation, weak correlation, moderate correlation, strong correlation and very strong correlation, respectively [47]. The results were considered significant if *p* < 0.05.

## 3. Results

**Figure 3:**
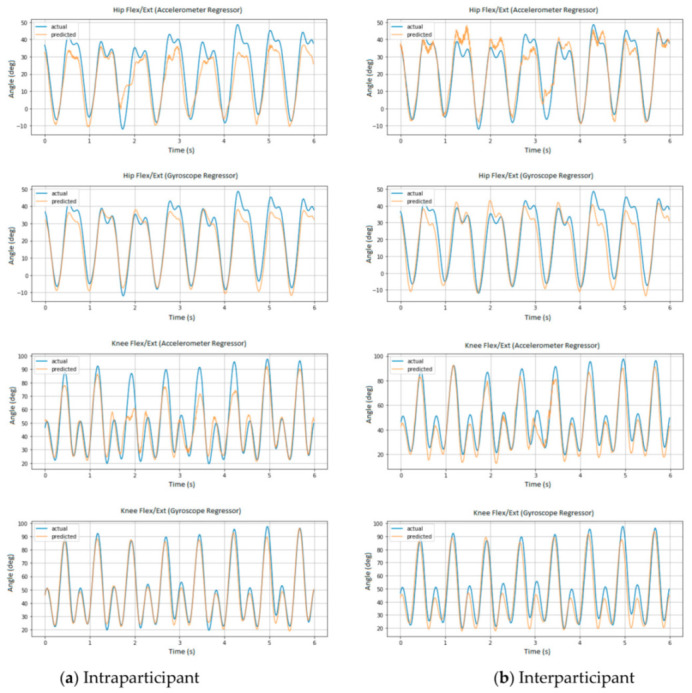
Actual and predicted angles in the sagittal plane of Participant 1 at six randomized intervals for (**a**) intraparticipant and (**b**) interparticipant models.

The actual and predicted angles in the sagittal plane of Participant 1 at typical 6 s intervals for intraparticipant and interparticipant models are presented in Figure 3.

### 3.1. Intraparticipant CNN Model

The average (standard deviation) R^2^, RMSE, NRMSE, STD, and NSTD of the accelerometer and gyroscope regressors for the knee and hip joint angle predictions are presented in Table 3a. In the intraparticipant CNN model, the average (standard deviation) R^2^, RMSE, NRMSE, STD, and NSTD for predicting the joint kinematics of both the knee and hip using the gyroscope regressor were found to be significantly better than those using the accelerometer regressor (see Table 3a).

### 3.2. Interparticipant CNN Model

The average (standard deviation) R^2^, RMSE, NRMSE, STD, and NSTD of the accelerometer and gyroscope regressors for the knee and hip joint angle predictions are presented in Table 3b. The use of the gyroscope regressor improved the average (standard deviation) R^2^, RMSE, NRMSE, STD, and NSTD relative to the use of the accelerometer regressor, despite most of these improvements being statistically nonsignificant (see Table 3b). Only the difference in the prediction of average STD and NSTD of hip kinematics by the two models was found to be significant (see Table 3b).

## 4. Discussion

This study investigated the performance in predicting level-ground running kinematics of deep learning algorithms trained on kinematics data obtained from individuals running on a treadmill. The performance of accelerometer and gyroscopic data as regressors in kinematics prediction was compared. A CNN model was used to predict knee joint and hip joint angles in intraparticipant and interparticipant scenarios. To the best of our knowledge, this is the first study to use this deep learning approach technique to predict level-ground running kinematics.

The kinematic prediction for the knee joint angle was better than that for the hip joint angle in the sagittal plane in both intraparticipant and interparticipant scenarios. The average R^2^ of the knee joint angle prediction with both accelerometer and gyroscope regressors had strong correlations [47]. The average R^2^ of the hip joint angle prediction with both accelerometer and gyroscope regressors also had strong correlations [47]. For the intraparticipant scenarios, the gyroscope regressor yielded significantly better predictions than the accelerometer regressor did. The results for the average R^2^ of the knee joint angle prediction with the accelerometer and gyroscope regressors were strongly correlated [47]. The results for the average R^2^ of the hip joint angle prediction with the accelerometer and gyroscope regressors were strongly correlated [47]. Although the gyroscope regressor yielded more accurate predictions compared with the accelerometer regressor, these improvements were statistically significant in the intraparticipant scenario. When the intraparticipant and interparticipant results were combined, the average R^2^ of the knee joint angle predictions exhibited a stronger correlation than that for the hip joint angle predictions. The average NRMSE percentage was also lower for the knee joint angle prediction. Intraparticipant predictions were better than interparticipant predictions overall.

Predictions for level-ground running joint angle kinematics from the use of gyroscope regressors were significantly more accurate than those from the use of accelerometer regressors. Rhudy and Mahoney [41] and Mahoney and Rhudy [42] did not verify the capabilities and performance of accelerometer and gyroscope IMUs, but they reported that the performance of gyroscopes and accelerometers differed depending on the specific kinematics measured. These results demonstrate that using the gyroscope yielded better predictions of running kinematics compared with using the accelerometer for both knee and hip joint angles and in both intraparticipant and interparticipant scenarios. Consequently, the gyroscope may be considered a better regressor than the accelerometer in running kinematics prediction.

The performance differences between using the accelerometer versus the gyroscope might be due to the stiffness of the running surface and the flexibility of the treadmill. The treadmill is flexible and shock absorbent, thus absorbing impact forces to a larger extent than when running on level ground. Because data from treadmill running were used to train the CNN model, the stiffness of the treadmill surface may have affected the predictions of the accelerometer and the gyroscope regressors differently, contributing to the inferior performance of the accelerometer. The acceleration data collected by the accelerometers were possibly attenuated by the treadmill belt, which should be further investigated in a future study.

The present study is the first to use kinematics data from treadmill running for CNN training to make predictions of level-ground running kinematics. The model predicted knee and hip angles during running using intraparticipant and interparticipant data. In contrast with studies [5,35,36,37] that were limited to predict treadmill running kinematics, this study provides new insight into and inspiration for techniques for predicting level-ground running kinematics. The results for both the intraparticipant and interparticipant scenarios were strongly to very strongly correlated and indicated that level-ground running kinematics could be predicted by treadmill running kinematics. Nevertheless, more studies are required to further investigate predictions of kinematics in other running surfaces and elucidate the kinematics of real-world running.

The movement planes and the degrees of freedom being investigated should also be considered. The prediction of the knee joint angle was better than that of the hip joint angle in the sagittal plane, and the errors for knee joint angle predictions were also lower than those for hip joint angle predictions. Because the knee joint has less freedom of movement in the frontal plane and transverse planes, only sagittal plane data were used for the comparison of knee joint angle and hip joint angle predictions. These findings are similar to those of other studies reporting that consistent trajectories explain the lower prediction error for the knee joint angle [5,33,36]. Furthermore, the prediction results could be affected by noise artifacts due to the attachment and alignment of the IMU sensors and magnetic distortion in the motion lab [48]. Therefore, in terms of running kinematics predictions, the knee joint angle is more consistent and predictable than the hip joint angle in the sagittal plane. Hip joint angle data were also affected by offset problems during data collection, influencing the prediction results. Considering the movement planes, degrees of freedom, and alignment errors, the knee joint is particularly suitable for angular kinematic estimates made with the use of a single IMU.

Finally, the results from the leave-one-out scheme adopted in the interparticipant trials demonstrated that the CNN model successfully learned from the knee joint angle data. The gyroscope regressor consistently outperformed the accelerometer regressor, corroborating the findings of [5]. Furthermore, training with hip joint angle data resulted in poorer test results compared with training with knee joint angle data. However, the intraparticipant results indicate that a training set of 10 participants was insufficient for general kinematics predictions.

## 5. Limitations

The small sample size of 10 recreational runners was a limitation of this study in that it limited the generalizability of the findings. Future studies could recruit more participants, accelerating the project of establishing a universal model for predicting running kinematics. Another way for building a more representative model is by carefully selecting subjects with a balanced distribution of physical characteristics such as height, weight, age, and gender as in a study by Sun et al. [49]. In that study, gait data of ten subjects were chosen with a balanced distribution and used to build an ideal gait database, which was subsequently used in conjunction with a novel neural network controller for actuating their prosthetic knee design in a speed-adaptive manner. As illustrated in Figure 3, prediction results were occasionally suboptimal, especially for the accelerometer regressor, which were the results of more difficult cross-field and cross-subject prediction. A better training set could be obtained either by enrolling more subjects or carefully choosing a more representative group [49].

As Gholami et al.’s [5] architecture and parameters were fully adopted in the current study for comparison purposes, it is conceivable that fine tuning of the network and other hyperparameters could improve the prediction accuracy. This is especially true when considering the use of a powerful and deep network of four Conv1D with two dense layers, in which more optimal parameter values can be grid searched and validated.

Another key limitation of this study was that the ankle joint angle data collected were very unstable with substantial errors. Offset errors and abnormalities were observed for the ankle joint waveforms; therefore, the ankle joint angle kinematics data were removed from the analysis. Interference caused by the magnetic field in the laboratory was suspected to be the main cause. The interference is likely largest closest to the ground and would therefore primarily affect the ankle joint sensors [48]. Instability of foot-mounted sensors may also have contributed to the poor data quality. Improving the placement and fixation of the sensors could be a goal of future studies collecting ankle kinematics data.

Finally, different placements of sensors may affect the prediction performance. In this study, the IMU sensor used to collect kinematics data was placed on the anteromedial side of the right tibia. Further studies may attempt different placements of the single sensor on the lower extremities to investigate the best placement of the single sensor and obtain better predictions.

## 6. Conclusions

In this study, a CNN deep learning model was used to predict level-ground running kinematics by treadmill running kinematics measured by a single IMU sensor. The predictions of knee and hip angles in the sagittal plane were mostly with strong to very strong correlations. The predictions of knee angles were better than that of hip angles. The predictions from using the gyroscope regressor were significantly more accurate than using the accelerometer regressor. Future studies may investigate predictions of running kinematics on different fields by using deep learning approaches. Predictions of running kinematics on different fields may enhance the understanding of running-related injury on different running surfaces.

## Figures and Tables

**Table 1:** Summary of parameters used in the CNN network.

Network Layer	Output Shape	Other Parameters	Value/Type
Input	(60, 1)	Training data shape	X(N, 60, 1), Y(N, 1), where N is the total number of 60 sample frames (0.6 s) for training
Conv1D	(58, 50)	Kernel initializer	GlorotNormal (all layers)
Conv1D	(56, 50)	Loss function	Mean squared error
MaxPolling1D	(28, 50)	Number of filters	50 (first 2 Conv1D), 100 (last 2 Conv1D)
Conv1D	(26, 100)	Kernel size/Stride/Padding	3/1/No padding (all Conv1D)
Conv1D	(24, 100)	Pool size	2 (for MaxPooling1D)
Flatten	(2400)	Activation	ReLU for all Conv1D and first Dense layer, Linear for last Dense layer
Dense	(100)	Epochs/Batch size	50/512
Dense	(1)	Learning rate	0.001

**Table 2:** Definitions and formulas of the six evaluation parameters: (1) average correlation (R^2^), (2) average root mean squared error (RMSE), (3) average normalized root mean squared error (NRMSE), (4) average standard deviation (STD), (5) average normalized standard deviation (NSTD), and (6) average range of motion (ROM).

Symbol	Definition	Formula
R^2^	Coefficient of determination: Applied to all points of actual and predicted data values.	$R^{2}=1-\frac{\sum_{i}{\left(y_{i}-{\hat{y}}_{i}\right)}^{2}}{\sum_{i}{\left(y_{i}-\bar{y}\right)}^{2}}$ where {$y_{i}$} is the set of data points of the target signal, $\bar{y}$ is the mean of {$y_{i}$}, and ${\hat{y}}_{i}$ is the predicted value of $y_{i}$ in the regression.
ROM	Range of motion: Applied to all points of actual and predicted data values.	$\mathrm{ROM}=y_{\max}-y_{\min}$ where {$y_{i}$} is the set of data points of the target signal and $y_{\max}$ is the maximum and $y_{\min}$ is the minimum of {$y_{i}$}.
RMSE	Root mean squared error: Applied to data points at specific gait events: For knee: peak flexion during stance (i.e., the ‘small’ peak, see Figure 1); For hip: peak flexion (see Figure 1).	$\mathrm{RMSE}=\sqrt{\frac{\sum {\left(p_{i}-{\hat{p}}_{i}\right)}^{2}}{N}}$ where {$p_{i}$} is the set of data points at specific gait events of the target signal (i.e., either at peak knee flexion during stance or peak hip flexion), N is the total number of points in {$p_{i}$}, and ${\hat{p}}_{i}$ is the predicted value of $p_{i}$ in the regression.
NRMSE	Normalized root mean squared error: Normalized the RMSE with overall range of data values.	$\mathrm{NRMSE}=\frac{\mathrm{RMSE}}{\mathrm{ROM}}=\frac{\mathrm{RMSE}}{y_{\max}-y_{\min}}$ where {$y_{i}$} is the set of data points of the target signal and $y_{\max}$ is the maximum and $y_{\min}$ is the minimum of {$y_{i}$}.
STD	Standard deviation of the residuals: Calculated at points of specific gait events: For knee: peak flexion during stance (i.e., the ‘small’ peak); For hip: peak flexion.	Standard deviation of the set of residuals {$r_{i}\;|{\;r}_{i}=p_{i}-{\hat{p}}_{i}$} where {$p_{i}$} is the set of data points at specific gait events of the target signal (i.e., either at peak knee flexion during stance or peak hip flexion) and ${\hat{p}}_{i}$ is the predicted value of $p_{i}$ in the regression.
NSTD	Normalized standard deviation of the residuals: Applied to all points of actual and predicted data values.	$\mathrm{NSTD}=\frac{\mathrm{STD}}{\mathrm{ROM}}=\frac{\mathrm{STD}}{y_{\max}-y_{\min}}$ where {$y_{i}$} is the set of data points of the target signal and $y_{\max}$ is the maximum and $y_{\min}$ is the minimum of {$y_{i}$}.

**Table 3:** Average correlation (R^2^), average root mean squared error (RMSE), average normalized root mean squared error (NRMSE), average standard deviation (STD), average normalized standard deviation (NSTD), and range of motion (ROM) for predicting knee and hip kinematics using an accelerometer and a gyroscope. Paired sample *t*-tests were used to compare each parameter between using an accelerometer and a gyroscope, with statistical significance (*p*) (in bold for *p* < 0.05).

a. Intraparticipant model											
	R^2^		RMSE (°)		NRMSE (%)		STD (°)		NSTD (%)		ROM
	Mean (SD)	*p*	Mean (SD)	*p*	Mean (SD)	*p*	Mean (SD)	*p*	Mean (SD)	*p*	Mean (SD)
Knee_Accel	0.93 (0.043)	**0.009**	4.4 (2.0)	**0.025**	4.9 (1.8)	**0.023**	3.2 (1.1)	**0.019**	3.2 (1.1)	**0.037**	88.7° (17.0°)
Knee_Gyro	0.96 (0.012)		3.2 (1.0)		3.6 (0.9)		2.4 (0.6)		2.4 (0.6)		
Hip_Accel	0.85 (0.075)	**0.01**	7.1 (2.6)	**0.026**	10.8 (3.4)	**0.025**	5.2 (2.1)	**0.044**	5.2 (2.1)	**0.044**	64.5° (10.7°)
Hip_Gyro	0.90 (0.064)		5.2 (1.7)		8.0 (1.9)		4.1 (1.2)		4.1 (1.2)		
b. Interparticipant model											
	R^2^		RMSE (°)		NRMSE (%)		STD (°)		NSTD (%)		ROM
	Mean (SD)	*p*	Mean (SD)	*p*	Mean (SD)	*p*	Mean (SD)	*p*	Mean (SD)	*p*	Mean (SD)
Knee_Accel	0.90 (0.060)	0.18	7.7 (2.6)	0.26	8.8 (3.1)	0.30	4.0 (1.9)	0.15	4.6 (2.4)	0.15	88.7° (17.0°)
Knee_Gyro	0.92 (0.065)		6.3 (3.1)		7.4 (4.0)		3.0 (1.1)		3.4 (1.1)		
Hip_Accel	0.73 (0.348)	0.16	6.6 (2.3)	0.72	10.3 (3.8)	0.60	5.6 (1.5)	**0.002**	8.6 (1.8)	**0.002**	64.5° (10.7°)
Hip_Gyro	0.83 (0.160)		6.8 (3.4)		10.8 (5.9)		4.6 (1.4)		7.0 (1.6)		

## Data Availability

Data sharing not applicable.
